# Impact of Alternate *b*-Value Combinations and Metrics on the Predictive Performance and Repeatability of Diffusion-Weighted MRI in Breast Cancer Treatment: Results from the ECOG-ACRIN A6698 Trial

**DOI:** 10.3390/tomography8020058

**Published:** 2022-03-04

**Authors:** Savannah C. Partridge, Jon Steingrimsson, David C. Newitt, Jessica E. Gibbs, Helga S. Marques, Patrick J. Bolan, Michael A. Boss, Thomas L. Chenevert, Mark A. Rosen, Nola M. Hylton

**Affiliations:** 1 Department of Radiology, University of Washington, Seattle, WA 98195, USA; 2 Department of Biostatistics and Center for Statistical Sciences, Brown University, Providence, RI 02912, USA; jon_steingrimsson@brown.edu (J.S.); hmarques@stat.brown.edu (H.S.M.); 3 Department of Radiology and Biomedical Imaging, University of California, San Francisco, CA 94143, USA; dnewitt@sbcglobal.net (D.C.N.); jessica.gibbs@ucsf.edu (J.E.G.); nola.hylton@ucsf.edu (N.M.H.); 4 Center for Magnetic Resonance Research, Department of Radiology, University of Minnesota, Minneapolis, MN 55455, USA; bolan@cmrr.umn.edu; 5 Center for Research and Innovation, American College of Radiology, Philadelphia, PA 19103, USA; mboss@acr.org; 6 University of Michigan, Ann Arbor, MI 48109, USA; tlchenev@med.umich.edu; 7 University of Pennsylvania, Philadelphia, PA 19104, USA; mark.rosen@pennmedicine.upenn.edu

**Keywords:** breast cancer, diffusion-weighted MRI (DW-MRI), apparent diffusion coefficient (ADC), treatment response, repeatability, reproducibility, quantitative imaging biomarker alliance (QIBA)

## Abstract

In diffusion-weighted MRI (DW-MRI), choice of *b*-value influences apparent diffusion coefficient (ADC) values by probing different aspects of the tissue microenvironment. As a secondary analysis of the multicenter ECOG-ACRIN A6698 trial, the purpose of this study was to investigate the impact of alternate *b*-value combinations on the performance and repeatability of tumor ADC as a predictive marker of breast cancer treatment response. The final analysis included 210 women who underwent standardized 4-*b*-value DW-MRI (*b* = 0/100/600/800 s/mm^2^) at multiple timepoints during neoadjuvant chemotherapy treatment and a subset (n = 71) who underwent test–retest scans. Centralized tumor ADC and perfusion fraction (*f_p_*) measures were performed using variable *b*-value combinations. Prediction of pathologic complete response (pCR) based on the mid-treatment/12-week percent change in each metric was estimated by area under the receiver operating characteristic curve (AUC). Repeatability was estimated by within-subject coefficient of variation (wCV). Results show that two-*b*-value ADC calculations provided non-inferior predictive value to four-*b*-value ADC calculations overall (AUCs = 0.60–0.61 versus AUC = 0.60) and for HR+/HER2− cancers where ADC was most predictive (AUCs = 0.75–0.78 versus AUC = 0.76), *p* < 0.05. Using two *b*-values (0/600 or 0/800 s/mm^2^) did not reduce ADC repeatability over the four-*b*-value calculation (wCVs = 4.9–5.2% versus 5.4%). The alternate metrics ADC_fast_ (*b* ≤ 100 s/mm^2^), ADC_slow_ (*b* ≥ 100 s/mm^2^), and *f_p_* did not improve predictive performance (AUCs = 0.54–0.60, *p* = 0.08–0.81), and ADC_fast_ and *f_p_* demonstrated the lowest repeatability (wCVs = 6.71% and 12.4%, respectively). In conclusion, breast tumor ADC calculated using a simple two-*b*-value approach can provide comparable predictive value and repeatability to full four-*b*-value measurements as a marker of treatment response.

## 1. Introduction

As oncologic approaches move increasingly towards personalization of therapies, improved methods for early assessment of breast cancer response to neoadjuvant chemotherapy (NAC) are needed to enable timely modification of therapeutic regimens. Clinical breast examination and routine breast imaging with mammography and ultrasound remain the standard-of-care methods for monitoring patients undergoing NAC; however, because they primarily reflect changes in gross tumor size or morphology, their sensitivity to detect early cytotoxic effects is limited. However, functional imaging technologies can allow for a more specific evaluation of vascular, metabolic, biochemical, and molecular changes in breast tumors in response to treatment. These include magnetic resonance imaging (MRI), fluorodeoxyglucose (FDG) positron emission tomography (PET), molecular breast imaging, and ultrasound and optical imaging techniques as recently reviewed by Rauch et al. Quantification of alterations in water diffusion through diffusion-weighted MRI (DW-MRI) holds strong potential to detect early treatment-induced changes in tumor microstructure, cellularity, and cell membrane integrity [1]. Indeed, numerous breast DW-MRI studies have demonstrated the apparent diffusion coefficient (ADC) metric to be useful in discriminating responders and non-responders in breast cancer treatment [2,3,4,5,6,7,8], and its utility for predicting pathological complete response (pCR) to NAC was recently summarized in a meta-analysis of 15 studies with 1181 total breast cancer patients [9].

Approaches for breast DW-MRI vary widely across prior studies, both in terms of acquisition and interpretation, which has been emphasized as a limitation to translation of ADC as a clinical biomarker. Consensus recommendations from the European Society of Breast Radiology were recently published to facilitate standardization and to provide best practices for achieving an adequate signal-to-noise ratio while minimizing artifacts and distortions [10]. These recommendations are largely based on expert opinions; more data are needed to refine optimal methods for implementation of ADC as a quantitative imaging biomarker in breast cancer clinical trials. The choice of *b*-values (number and range) is known to influence calculated breast lesion ADC values and affect scan times, but the impact on the reliability and performance of ADC as an imaging marker is not well understood. The Quantitative Imaging Biomarkers Alliance (QIBA) of the Radiological Society of North America put forth a DWI Profile with acquisition and analysis specifications to support use of ADC as a robust quantitative biomarker [11], with a quantitative claim indicating that the 95% confidence interval of a 13% or larger measured change in the apparent diffusion coefficient (ΔADC) is a true change. The Profile’s imaging protocols derive from two test–retest studies [12,13] and indicate the number of different *b*-values to achieve the claim: ideally four, with a target of three *b*-values. Test–retest data indicating similar repeatability with the use of fewer *b*-values would allow for shorter acquisitions.

The ECOG-ACRIN Cancer Research Group A6698 multicenter trial, performed as a substudy to the ongoing Investigation of Serial Studies to Predict Your Therapeutic Response with Imaging and Molecular Analysis 2 (I-SPY 2) trial, was designed to validate the performance of breast tumor ADC measures for predicting pathologic response to NAC using a generalizable standardized approach and a four-*b*-value acquisition. Results from the trial’s primary analysis confirmed mid-treatment percent change in tumor ADC (after 12 weeks of chemotherapy) to be significantly predictive of pCR (overall AUC = 0.60, 95% CI 0.52–0.68), with increased accuracy obtained by accounting for breast cancer subtype (AUC = 0.72, 95% CI 0.61–0.83) [14]. A second aim of the trial assessed variability between repeated scans using a test–retest ‘coffee break’ design, which showed that excellent repeatability and reproducibility of breast tumor ADC measures could be achieved in a multi-institution setting using a standardized protocol and QA procedure [12]. As a secondary analysis of the same study cohort, the purpose of this study was to investigate the impact of alternate *b*-value combinations for calculating ADC on both predictive performance and measurement repeatability.

## 2. Materials and Methods

### 2.1. Study Participants

**Figure 1:**
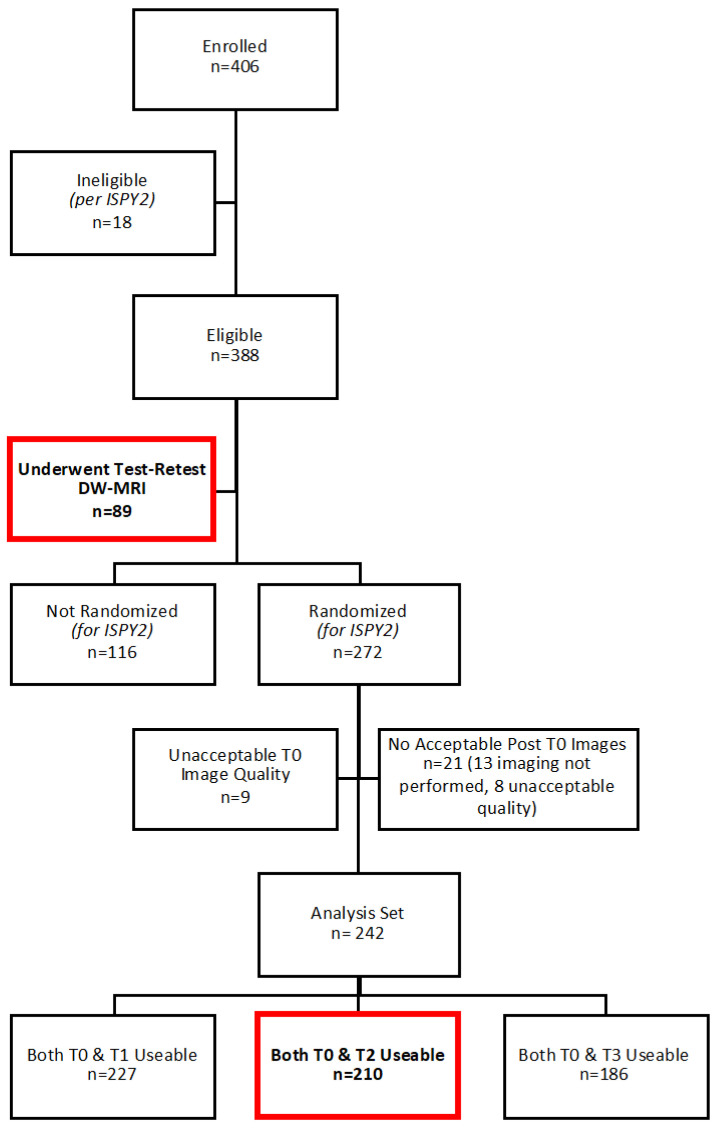
Consolidated Standards of Reporting Trials diagram summarizing ACRIN 6698 study patient enrollments. (Timepoints: T0, pre-treatment; T1, early treatment; T2, mid-treatment; and T3, post-treatment.) Subjects evaluated in this secondary analysis are indicated by bold text/red boxes.

In this prospective, Health Insurance Portability and Accountability Act-compliant, multi-institution study, consecutive subjects enrolled in I-SPY 2 at sites meeting DW-MRI qualification requirements were also co-enrolled in the ACRIN 6698 imaging trial (ClinicalTrials.gov: NCT01564368 [15,16]). Eligibility for I-SPY 2 included women ≥ 18 years of age with invasive breast tumors ≥2.5 cm in size by clinical exam or imaging, and intent to undergo neoadjuvant chemotherapy. Subjects with evidence of distant metastasis were excluded, and those determined to have low-risk disease (i.e., hormone receptor (HR)+/HER2-negative/MammaPrint low) did not proceed to the treatment arm of I-SPY 2. Both the I-SPY 2 and ACRIN 6698 protocols were approved by institutional review boards at all participating sites (listed in the Supplemental Material), and all subjects gave written informed consent using a single combined consent form. ACRIN 6698 was powered to achieve 160 evaluable subjects to adequately test whether changes in tumor ADC during treatment were predictive of pCR; thus, target enrollment was set for 404 participants to account for dropout and expected I-SPY 2 screen fails (more detail provided in the Supplemental Material and [16]). A Consolidated Standards of Reporting Trials flow diagram describing the inclusion and exclusion of trial subjects is provided in Figure 1.

**Figure 2:**
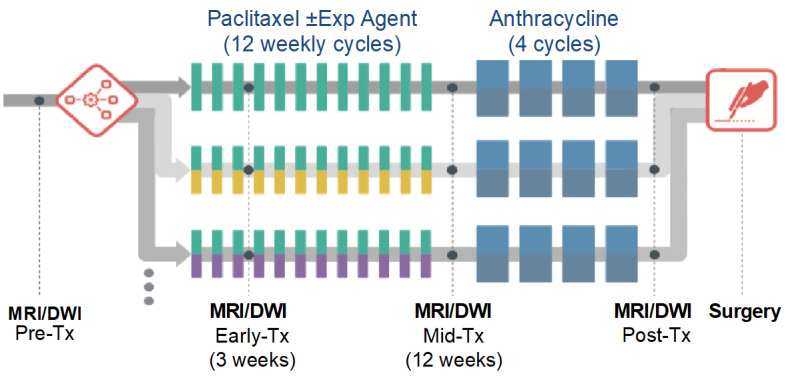
ECOG-ACRIN A6698 and parent I-SPY 2 Trial study schema. Exp, experimental; Tx, treatment. Reprinted with permission from Partridge et al. Radiology 2018; 289(3): 618–627.

Per the I-SPY 2 protocol, MRI examinations with DW-MRI were performed at pre-treatment, early treatment (after 3 weekly doses of paclitaxel/taxane-based therapy), mid-treatment (12 weeks, between taxane and anthracycline regimens), and post-treatment (after all chemotherapy) timepoints, prior to surgery (Figure 2). For the ACRIN 6698 Trial, an advanced 4-*b*-value DW-MRI sequence was added at each of the MRI timepoints, acquired prior to contrast injection. Test/retest repeatability scans were performed on a subset of patients at pre- or early treatment MRI exams. Individual sites were limited to 14 test/retest patients to better balance the accrual across different MRI scanner manufacturers and field strengths.

The study sample was previously published in the primary analysis evaluating the predictive value of changes in tumor ADC [14], and a subset of 71 subjects were described in two publications evaluating the repeatability of tumor ADC [12] and histogram measures [17]. This secondary analysis assesses the relative performance of various alternative ADC metrics. Data generated or analyzed during the study are available through ECOG-ACRIN, and all images and associated study metadata will be available on The Cancer Imaging Archive (TCIA) website [18], planned for public release in Spring 2022.

### 2.2. MRI Acquisition

The A6698 imaging protocol and the DW-MRI quality assurance process have been previously reported [12,14]. Briefly, all MRI examinations included standardized T2-weighted, DW-MRI, and dynamic contrast-enhanced (DCE)-MRI sequences (acquisition parameters are given in Supplemental Table S1). DW-MRI was acquired prior to DCE-MRI in the axial orientation with diffusion gradients in three orthogonal directions using multiple *b*-values (0, 100, 600, and 800 s/mm^2^), with a single-shot, diffusion-weighted, spin-echo echo-planar imaging sequence with parallel imaging (reduction factor ≥ 2) and fat suppression. Required scan parameters were TR ≥ 4000 ms, TE minimum (50–100 ms), flip angle 90°, field of view 300–360 mm, acquired matrix 128 × 128 to 192 × 192, and scan time ≤ 5 min. The acquired resolution was 1.7–2.8 mm in-plane with a 4–5 mm slice thickness. No respiratory triggering or other motion compensation methods were used. Test and retest DW-MRI scans for a given patient were performed in the same imaging examination, at either the pre-treatment (preferred) or early treatment timepoint. The patient was positioned normally (prone) and scanned with initial localization, T2W, and DW-MRI acquisitions. They were then removed from the scanner and taken off the scanner bed, then repositioned as before. The full ACRIN 6698 protocol was then performed, consisting of localization, T2W, DW-MRI, and DCE acquisitions.

Prior to study participation, all sites were required to pass quality control testing consisting of DW-MRI phantom scanning and submission of two in vivo DW-MRI cases acquired using the multi-*b*-value protocol (previously described in the Appendix of [14]). In vivo images were reviewed for absence of substantial artifacts, homogenous fat suppression, and adequate signal-to-noise ratio.

### 2.3. ADC Measurements

Centralized image analysis was performed by trained researchers at the University of California, San Francisco, blinded to pathologic outcomes (final review performed by J.E.G. with over 10 years of quantitative breast MR analysis experience), using custom software tools developed with IDL (Exelis Visual Information Solutions, Boulder, Colorado) as previously described [14]. Evaluability on DW-MRI was first determined based on an acceptable signal-to-noise ratio, an acceptable degree of fat suppression, an absence of detrimental artifacts and distortions, and partial volume averaging. ADC maps were calculated using the classic monoexponential decay model [19] with linear least squares fitting of the log of the signal vs. *b*-value using all *b*-values (0/100/600/800 s/mm^2^), as in the primary analysis [14], and additionally for a variety of alternate *b*-value combinations including ADC_fast_ (using *b* = 0/100 s/mm^2^) emphasizing microcirculation/perfusion, ADC_slow_ (using *b* = 100/600/800 s/mm^2^) minimizing perfusion influence [20], and two-*b*-value estimates (0/600, 0/800, 100/600, and 100/800 s/mm^2^) to reduce scan times and increase efficiency.

Perfusion fraction, *f_p_*, defined as the fraction of the total signal at *b* = 0 s/mm^2^ not accounted for by ADC_slow_, was also estimated from the 4-*b*-value data as:

*f_p_* = [S(0)–S_0slow_]/S(0) (1)

where S(0) is the measured signal at *b* = 0 s/mm^2^ and S_0slow_ is the *b* = 0 intercept of the mono-exponential fit for ADC_slow_ [21].

This is a simplified analysis that follows the original approach of Le Bihan et al. [22] and assumes that perfusion effects are negligible at *b* = 100 s/mm^2^. The acquisition used for this study did not sample enough *b*-values for use with advanced fitting strategies to robustly separate diffusion from perfusion effects [23,24]. Although beyond the primary scope of the A6698 trial, formal IVIM modeling can give unique insights by accounting for the microvascular contribution to the DWI signal. By more densely sampling at very low *b*-values to accurately measure the signal decay related to microcirculation followed by a biexponential fit of the data, IVIM analysis enables separate characterization of the vascular and tissue components of the diffusion signal, including the perfusion fraction (*f*), the pseudo-diffusion rate, reflecting capillary blood flow (D*), and true tissue diffusion (D_t_) [22].

**Figure 3:**
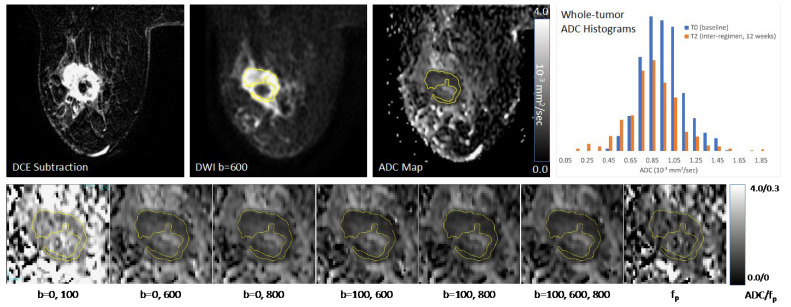
Sample images and diffusion maps from one non-pCR patient at baseline. The top row shows matched slices from the dynamic contrast-enhanced (DCE) early subtraction image, the diffusion-weighted image (DWI), and the quantitative apparent diffusion coefficient (ADC) map. Histograms are shown for the whole-tumor ADC values for this pre-treatment (T0) scan and the patient’s mid-treatment timepoint (T2) MRI. The subject showed a steady decrease in tumor volume both on DCE and DWI, but no recovery towards normal tissue ADC values was observed for the mean ADC. The bottom row shows the tumor region ADC maps for all *b*-value combinations (units 10^−3^ mm^2^/s) plus the *f_p_* map.

Tumor was identified on post-contrast DCE subtraction images and then localized on DW-MRI. Multi-slice, whole-tumor regions-of-interest (ROIs) were manually defined by selecting regions with hyperintensity on high *b*-value DW-MRI (*b* = 600 or 800 s/mm^2^) and a relatively low ADC while avoiding adjacent adipose and fibroglandular tissue, biopsy clip artifacts, and regions of high T2 signal (e.g., seroma and necrosis). For large and multicentric/multifocal disease, all disease regions were included in the ROI and several distinct contours could be drawn on multiple slices to cover the entire tumor region as depicted in the DCE images, without including intervening stroma. All voxels from separate contours were then combined into a single composite ROI to represent the entire tumor and the mean ADC was calculated. Tumor ROIs were redefined for each treatment timepoint, referencing the lesion location on prior exams. In tumors without residual enhancement on DCE after treatment, ROIs were defined in the same tissue region as the prior examination. ROIs were the same ones used in the primary analysis (not redrawn for this study). ROIs were then propagated to the various ADC and *f_p_* maps. An example of serial ADC quantitation in a study patient is shown in Figure 3.

### 2.4. Reference Standard for Pathologic Response

Histopathologic analysis was performed at study sites by institutional pathologists (blinded to MRI measures) according to the I-SPY 2 TRIAL protocol using the Residual Cancer Burden system [25,26]. Following U.S. Food and Drug Administration rationale and guidelines [27], pathologic complete response (pCR) was the reference standard for determining response to neoadjuvant chemotherapy in our study, defined and reported as no residual invasive disease in either breast or axillary lymph nodes after neoadjuvant therapy (ypT0/is, ypN0). Subjects were categorized as pCR or non-pCR based on postsurgical histopathology.

### 2.5. Statistical Analysis

Pearson’s correlation coefficients (r) were used to estimate correlations between pretreatment metrics measured using different *b*-value combinations. Mid-treatment/12-week percent change from pre-treatment values was calculated for each metric, and performance for predicting pCR was evaluated by receiver operating characteristic (ROC) curves and associated areas under the curve (AUC). A Delong’s non-inferiority test, using a pre-specified non-inferiority margin of 0.02, was used to compare AUCs of the ADC estimates using 2-*b*-value combinations of 0/600, 0/800, 100/600, and 100/800 s/mm^2^ to the reference ADC metric using all four *b*-values. A non-inferiority test was used because in the case of similar prediction accuracy an ADC metric using a 2-*b*-value combination might be preferred over the 4-*b*-value combination due to reduced imaging time and simplified breast DW-MRI acquisition.

A Delong’s test of superiority was used to compare the AUCs of the alternate metrics of ADC_fast_ and ADC_slow_ (calculated with *b*-values of 0/100 and 100/600/800 s/mm^2^, respectively) and *f_p_* to the reference ADC metric using all four *b*-values. Finally, a Delong’s test of superiority was also used to compare AUCs between a multivariate model, which included the potentially complementary metrics ADC_fast_ and ADC_slow_, and the reference ADC metric. Analyses were performed for all cancers and within the HR+/HER2− subtype (identified in the primary analysis as the subtype for which mid-treatment change in ADC was most predictive [14]). To account for multiplicity, we used a hierarchal testing procedure [28] that only performed a hypothesis test within the HR/HER2 subtype when the corresponding test for all cancers was rejected. A Bonferroni correction was used to account for the multiple hierarchal testing procedures and all hypothesis tests were therefore interpreted using a significance level of 0.05/7 = 0.0071.

Repeatability of the different ADC metrics from test–retest acquisitions was evaluated using within-subject coefficient of variation (wCV) and limits of agreement were calculated in conformance with QIBA metrology guidelines [17,29]. Analyses were performed using SAS/STAT v9.4 (SAS Institute, Cary, NC, USA) and R v4.0.2 (R Foundation for Statistical Computing, Vienna, Austria).

## 3. Results

### 3.1. Participant Characteristics

Of the 406 consecutive women enrolled in the ACRIN 6698 trial at 10 institutions, 196 were excluded from this secondary analysis: 134 (33.0%) were not ultimately randomized to treatment on I-SPY 2 and 62 (15.3%) had missing or non-evaluable DW-MRI scans at pre- and/or mid-treatment timepoints (Figure 1); further details were previously reported [14]. Therefore, 210 women were evaluated (median age 48, IQR 40 to 56, range 25 to 77 years). A majority had grade III breast cancer (n = 146, 69.5%) and either the HR+/HER2− (n = 88, 41.9%) or HR−/HER2− (triple-negative, n = 65, 31.0%) subtype, and 70 (33.3%) achieved pCR (Table 1). Multiple MRI vendor systems were represented, and the majority of subjects (148/210; 70.5%) were imaged at 1.5 T (Supplemental Table S2).

Additionally, from the full cohort of 406 ACRIN 6698 patients, a subset of 89 patients consented to the test/retest substudy (Figure 1), of which 71 patients from 8 institutions (median age 46, range 27 to 71 years) had analyzable repeat DW-MRI scans (Table 1). Three of the eighty-nine patients (3.4%) were excluded for protocol deviations and fifteen (16.9%) for image quality issues in one (n = 7) or both (n = 8) of the test/retest DW-MRI acquisitions, as previously described [12].

#### 3.1.1. Correlation between Metrics

Pre-treatment tumor ADC measures calculated with different *b*-value combinations were highly correlated (r ≥ 0.92), with the exception of ADC_fast_ using the low *b*-value combination of 0/100 s/mm^2^ (r = 0.56–0.71; Table 2). Perfusion fraction, *f_p_*_,_ exhibited only weak correlations with the ADC metrics (r < 0.20) except ADC_fast_ (r = 0.75).

#### 3.1.2. Association with Pathologic Response

**Figure 4:**
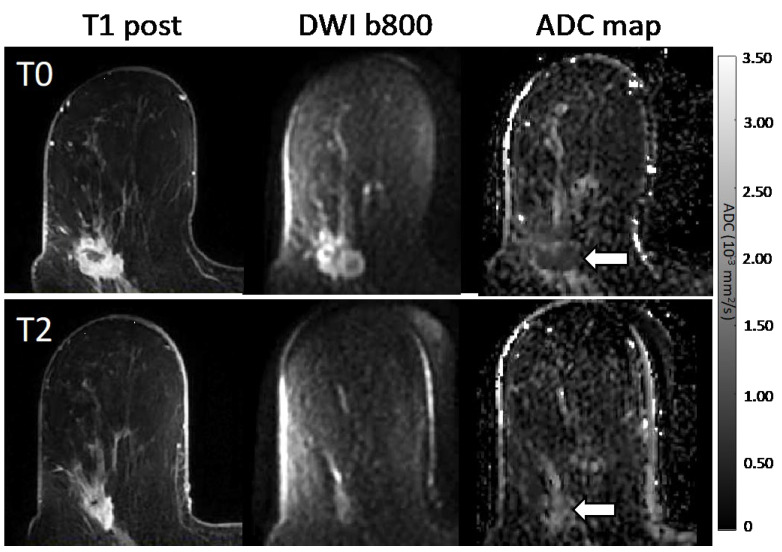
Serial MR images in a 58-year-old patient with a 3.7 cm HR+/HER2− high-grade invasive ductal carcinoma with pCR outcome. Shown are representative matched slices from the dynamic contrast-enhanced (DCE) early post-contrast scan, diffusion-weighted image (DWI; *b* = 800 s/mm^2^), and quantitative apparent diffusion coefficient (ADC) map for MRI examinations at the pre-treatment (T0, top) and mid-treatment (T2, bottom) timepoints. Between T0 and T2, the subject showed a decrease in tumor volume on DCE from 7.0 cc to 1.2 cc between timepoints, and the mean tumor ADC increased from 1.00 to 1.81 × 10^−3^ mm^2^/s.

**Figure 5:**
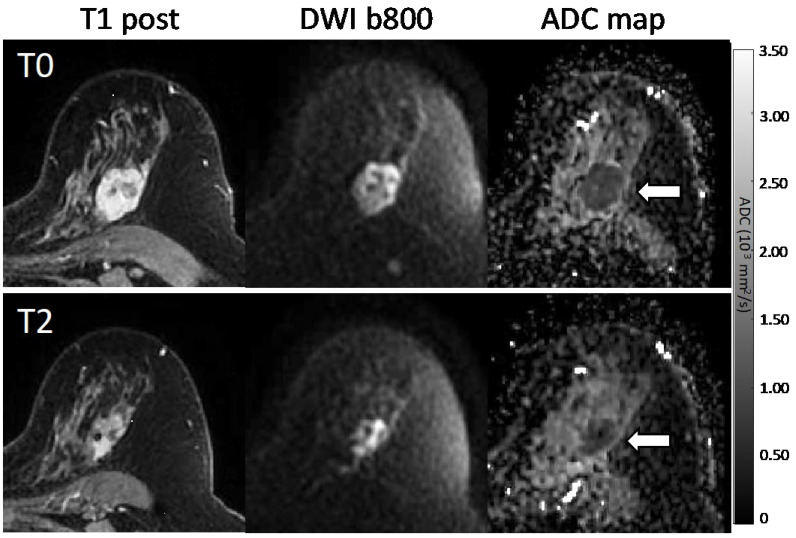
Serial MR images in a 57-year-old patient with a 3.2 cm HR+/HER2− high-grade invasive ductal carcinoma with non-pCR outcome. Shown are representative matched slices from the dynamic contrast-enhanced (DCE) early post-contrast scan, diffusion-weighted image (DWI; *b* = 800 s/mm^2^), and quantitative apparent diffusion coefficient (ADC) map for MRI examinations at the pre-treatment (T0, top) and mid-treatment (T2, bottom) timepoints. Between T0 and T2, the subject showed a decrease in tumor volume on DCE from 12.7 cc to 5.2 cc, while the mean tumor ADC remained unchanged at 0.92 × 10^−3^ mm^2^/s.

**Figure 6:**
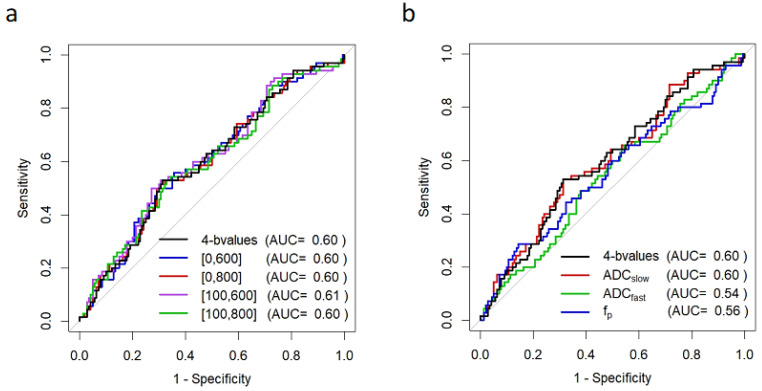
ROC curves for prediction of pCR based on mid-treatment change in DW-MRI metrics. Shown are ROC curves using percent change in (**a**) tumor ADC calculated using alternate *b*-value combinations of *b* = 0/600, 0/800, 100/600, and 100/800 s/mm^2^, and (**b**) alternate DW-MRI metrics of ADC_fast_ (using *b* = 0/100 s/mm^2^), ADC_slow_ (using *b* = 100/600/800 s/mm^2^), and perfusion fraction, *f_p_*. Percent change in the reference ADC calculated using all *b*-values (0/100/600/800 s/mm^2^) is shown on each plot for comparison (black). Curves reflect data for N = 210 women for all metrics except *f_p_* (N = 209; mid-treatment *f_p_* could not be accurately calculated in one subject with a very small lesion). Abbreviations: AUC, area under the ROC curve.

In general, a greater pre- to mid-treatment increase in tumor ADC was associated with pCR for all *b*-value combinations (Table 3). Examples of ADC response are shown in patients with pCR (Figure 4) and non-pCR (Figure 5) outcomes. Compared with the reference percent change in ADC using all *b*-values (0/100/600/800 s/mm^2^) with AUC = 0.60, 95% CI 0.52–0.68, two-*b*-value estimates (0/600, 0/800, 100/600, and 100/800 s/mm^2^) provided comparable performance, and the choice of the maximum *b*-value (600 vs. 800 s/mm^2^) did not affect diagnostic performance (AUCs 0.60–0.61; Figure 6, Table 3). Non-inferiority was confirmed for *b*-value combinations of 0/600, 0/800, 100/600, and 100/800 s/mm^2^ (all *p* < 0.05 after accounting for multiple comparisons).

**Figure 7:**
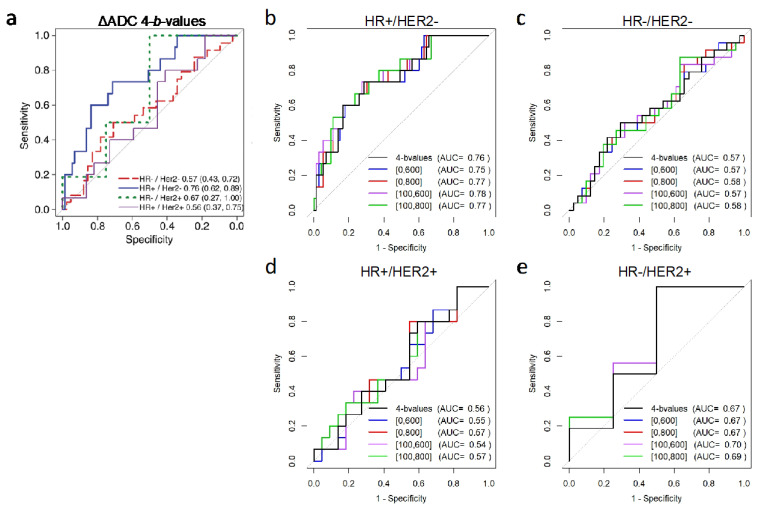
Subtype-based ROC analysis for prediction of pCR based on mid-treatment change in ADC. Shown are (**a**) ROC curves for percent change in tumor ADC calculated using all four *b*-values (0/100/600/800 s/mm^2^) stratified by cancer subtype. Additionally shown are ROC curves for two versus four *b*-value ADC calculations separately for participants with cancer subtypes of (**b**) HR+/HER2− (N = 88), (**c**) HR−/HER− (triple-negative; n = 65), (**d**) HR+/HER2+ (n = 37), and (**e**) HR−/HER2+ (N = 20). In panels (**b**–**e**), the ROC curve for the reference four-*b*-value ADC is shown in black. Abbreviations: AUC, area under the ROC curve; HR, hormone receptor; HER2, human epidermal growth factor receptor 2. Figure 7a reprinted from Partridge et al. Radiology 2018; 289(3): 618–627.

Stratification by cancer subtype demonstrated similar predictive value for ADC using two versus four-*b*-value combinations within each subtype, with non-inferiority confirmed in the HR+/HER2− subtype where ADC was most predictive of pCR (two-*b*-value AUCs = 0.75–0.78 versus four-*b*-value AUC = 0.76, *p* < 0.05 after multiplicity correction; Table 4, Figure 7).

Separating ADC into fast (*b*-values: 0/100 s/mm^2^) and slow (*b*-values: 100/600/800 s/mm^2^) components did not increase predictive performance (AUC = 0.54, 95% CI 0.46–0.62, *p* = 0.08; AUC = 0.60, 95% CI 0.52–0.68, *p* = 0.81, respectively; Figure 6b, Table 3), nor did a multivariate model combining ADC_fast_ and ADC_slow_ (AUC = 0.60, 95% CI 0.52–0.68, *p* = 0.99). Perfusion fraction, *f_p_*_,_ tended to decrease slightly more in the pCR group, but was not more predictive than the reference ADC metric (AUC = 0.56, 95% CI 0.47–0.64, *p* = 0.46; Figure 6b).

#### 3.1.3. Test–Retest Repeatability

Comparisons of test and retest DW-MRI measurements were performed in 71 patients. Test and retest ADC values were similar for the four-*b*-value combination (mean (SD) ADC: 1.16 (0.32) and 1.17 (0.31) × 10^−3^ mm^2^/s respectively) with minimal evidence of bias over tumor ADC values ranging from 0.80 to 2.62 × 10^−3^ mm^2^/s. We also observed minimal bias and no trend in the ADC difference with mean ADC for alternate *b*-value combinations of 0/600, 0/800, 100/600, 100/800, 0/100 (ADC_fast_), and 100/600/800 (ADC_slow_) s/mm^2^. Further, wCVs for ADC were comparable across all *b*-value combinations, ranging from 4.94% to 6.71% (Table 5). ADC_fast_ (*b* = 0/100 s/mm^2^) demonstrated the widest limits of agreement (−0.312 to 0.331 × 10^−3^ mm^2^/s) and highest wCV (6.71% (95% CI 5.76–8.02)), suggesting reduced repeatability versus other *b*-value ADC metrics. *f_p_* demonstrated poor repeatability compared with the ADC metrics (wCV = 12.37% (10.63–14.80)). The adjusted QIBA breast DWI claim from these data (excluding ADC_fast_) would be a 16.8% ΔADC having 95% confidence of being a true change, across all *b*-value pairs in this study.

## 4. Discussion

Our study found that two-*b*-value combinations for measuring ADC changes were no less predictive of treatment outcome than four-*b*-value combinations in women undergoing neoadjuvant chemotherapy for breast cancer, with pathologic complete responders demonstrating greater increases in tumor ADC after 12 weeks of therapy than non-complete responders. We also found that using fewer *b*-values did not reduce the repeatability of ADC as a quantitative breast cancer marker.

Using data from the ECOG-ACRIN A6698 multicenter trial, we evaluated ADC calculations with varying *b*-value combinations that potentially probe different underlying biological properties, provide different sampling of signal decay as a function of *b*-value and have different requirements for scan time. These ADC metrics demonstrated AUCs ranging from 0.54 to 0.61 for predicting pCR at mid-treatment. Using fewer than four *b*-values did not negatively impact performance (AUCs = 0.60–0.61) versus the benchmark established from the trial’s primary analysis using all *b*-values (AUC = 0.60), with the exception of the low *b* = 0/100 s/mm^2^ combination (AUC = 0.54). Moreover, using only higher non-zero *b*-values to minimize perfusion effects (i.e., measuring the slow ADC component) did not further improve pCR prediction (AUC = 0.60). Our results therefore suggest that for breast tumor diffusion measurements, an acquisition using only two *b*-values (e.g., 0/600 or 0/800 s/mm^2^) is sufficient to implement ADC as a reliable quantitative imaging marker in breast cancer clinical trials. This result can reduce the burden on sites (and their patients) implementing the QIBA DWI Profile by decreasing the target ideal number of *b*-values to two, with a nominal increase in the wCV and the resultant claim.

A growing number of studies have explored ADC for early identification of treatment efficacy in breast cancer; however, to date these studies have varied in study design and DW-MRI approach and typically comprise smaller, single-center datasets (average of 75 patients) [9]. The primary analysis from our large-scale multicenter prospective trial extends this work to provide a more generalizable assessment of ADC as a quantitative biomarker to predict pathologic response to NAC. This secondary analysis further demonstrates the robustness of ADC as a predictive marker in breast cancer treatment, as variable measurement approaches did not notably affect diagnostic performance. Our findings build on prior studies investigating optimal *b*-value combinations for breast tumor characterization [30,31,32,33,34] and support using the two-*b*-value combination of 0/800 s/mm^2^ as proposed in recent breast DW-MRI consensus recommendations [10], allowing for minimization of DW-MRI scan time and improved suitability for abbreviated breast MRI protocols or for strategies to utilize the extra time to increase spatial resolution.

Results of this study are important for developing guidelines for standardized and accurate use of ADC for identification of therapeutic effects in breast cancer clinical trials. The QIBA group recently added a breast claim to their DW-MRI profile defining error rates for breast tumor ADC calculations [11] based primarily on the A6698 trial test–retest data [12]. However, the confidence intervals were only known for the very narrow approach using all four *b*-values (0/100/600/800 s/mm^2^) used in the primary analysis for ADC calculation. There remains a dearth of test–retest data in the literature with an adequate sample size to expand upon these recommendations and allow for more flexible acquisition and analysis approaches; QIBA investigators suggest that estimates of precision should be based on a sample size of at least N = 35 to provide true 95% confidence intervals for a patient’s quantitative imaging biomarker measurement and for changes in the biomarker over time [29,35]. Results of this study provide new evidence that will allow QIBA to incorporate performance metrics for a wider range of breast DW-MRI protocols, particularly two-*b*-value acquisitions and greater specification of the target maximum *b*-value (800 s/mm^2^). Simplifying the approach to ADC calculation without compromising performance would likely facilitate broader implementation in multicenter trials. The alternate approaches and findings reported here align well with the consensus recommendations of the EUSOBI for standardization of breast DW-MRI (including use of two or more *b*-values, with a maximum *b* = 800 s/mm^2^ [10]) and thus will enable investigators to develop protocols conforming to both the QIBA and EUSOBI Breast DW-MRI guidelines for accuracy and standardization of breast ADC measures, respectively.

Our study has several limitations. The trial was not powered for these secondary analyses, and larger sample sizes may be needed to identify subtle differences in diagnostic performance. Additionally, we primarily explored only monoexponential ADC modeling over a limited *b*-value range (0–800 s/mm^2^), while sampling at higher diffusion weightings (e.g., *b* ≥ 1500 s/mm^2^) and utilizing more advanced non-Gaussian or multi-exponential DW-MRI modeling may better characterize the tissue microstructure and improve predictive performance [36,37,38,39]. While we explored perfusion fraction as a potential metric, the *b*-values were not optimized for IVIM analysis and extending acquisitions to more than four *b*-values would be needed to accurately characterize this parameter. In addition, measurements were calculated by averaging over all voxels in the manually defined whole-tumor ROIs. Alternate analytic approaches are under investigation to improve our ability to detect changes in tumor cellularity, including radiomics and histogram-based analyses and characterization of the ‘worst’ hot-spot (i.e., lowest ADC) tumor subregion. Furthermore, while the predictive performances achieved by ADC measures alone in this study were relatively modest, the ACRIN 6698 primary analysis demonstrated that higher AUCs could be achieved through multivariable modeling to incorporate important clinical characteristics, such as tumor HR/HER2 subtype [14], and additional consideration of other biologic factors (e.g., age, breast density, histopathology) could further improve predictive accuracy. While this analysis focuses on prediction of pCR, in future work it will be informative to test the value of ADC for predicting recurrence-free survival as study follow-up data mature.

In conclusion, these secondary analyses of ADC as a quantitative imaging biomarker in breast cancer treatment suggest that simple whole-tumor ADC measures using a two-*b*-value acquisition (e.g., 0/800 s/mm^2^) provide comparable accuracy and repeatability to four-*b*-value acquisitions and other two-*b*-value combinations. Extension of these findings to alternative DW-MRI approaches incorporating spatial variation (histogram analyses, radiomics, etc.) or non-Gaussian diffusion models remains to be investigated. This study contributes important additional data to inform and expand QIBA and other guidelines on optimal implementation of breast DW-MRI and utilization of ADC as a marker of response in breast cancer trials.

## Figures and Tables

**Table 1:** Patient characteristics.

	Analysis Set N = 210		Test–Retest Set N = 71	
**Age (years)**				
Mean ± Std Dev	48.3 ± 10.7		46.3 ± 11.1	
Median (Min–Max)	48.0	(25.0–77.0)	46.0	(27.0–71.0)
**Race, n (%)**				
White	153	(72.9)	53	(74.6)
Black	21	(10.0)	8	(11.3)
Asian	14	(6.7)	7	(9.9)
Native Hawaiian/Pacific Islander	1	(0.5)	0	0
Not Reported/Unknown	21	(10.0)	3	(4.2)
**Ethnicity, n (%)**				
Hispanic or Latino	20	(9.5)	5	(7.0)
Not Hispanic or Latino	137	(65.2)	62	(87.3)
Not Reported/Unknown	53	(25.2)	4	(5.6)
**HR/HER2 Subtype, n (%)**				
HR−/HER2− (TN)	65	(31.0)	17	(23.9)
HR+/HER2−	88	(41.9)	31	(43.7)
HR−/HER2+	20	(9.5)	9	(12.7)
HR+/HER2+	37	(17.6)	8	(11.3)
Missing	0		6	(8.5)
**MRI Longest Diameter at Baseline (cm)**				
Mean (Std Dev)	4.2 ± 2.3		4.5 ± 2.3	
Median (Min–Max)	3.5	(0.4–15.0)	3.9	(0.4–13.2)
Missing, n (%)	0		8	(11.3)
**Lesion Type, n (%)**				
Single mass	81	(38.6)	25	(35.2)
Single NME	9	(4.3)	7	(9.9)
Multiple masses	109	(51.9)	35	(49.3)
Multiple NME	11	(5.2)	4	(5.6)
**Tumor Grade, n (%)**				
I (Low)	5	(2.4)	2	(2.8)
II (Intermediate)	58	(27.6)	16	(22.5)
III (High)	146	(69.5)	48	(67.6)
N/A	1	(0.5)	4	(5.6)
Missing	0		1	(1.4)
**Pathologic Response, n (%)**				
Non-pCR	140	(66.7)	42	(59.2)
pCR	70	(33.3)	17	(23.9)
Missing	0		12	(16.9)

Abbreviations: NME, non-mass enhancement; HR, hormonal receptor; HER2, human epidermal growth factor receptor 2; pCR, pathologic complete response.

**Table 2:** Correlations between pre-treatment tumor ADC and *f_p_* metrics using different *b*-value combinations.

Pearson’s Correlation Coefficients							
	All *b*-Values	0/600	0/800	100/600	100/800	ADC_fast_	ADC_slow_
0/600	0.99						
0/800	0.99	0.95					
100/600	0.98	0.98	0.94				
100/800	0.97	0.92	0.99	0.94			
ADC_fast_	0.67	0.71	0.67	0.57	0.56		
ADC_slow_	0.98	0.94	0.99	0.96	1.00	0.57	
*f_p_*	0.13	0.19	0.14	0.06	0.03	0.75	0.04

**Table 3:** Relative performance of mid-treatment percent change in alternate tumor ADC, *f_p_* metrics for predicting pCR.

Mid-Treatment Δ	pCR (N = 70) Mean ± SD (%)	Non-pCR (N = 140) Mean ± SD (%)	AUC [95% CI]	*p*-Value
ΔADC: All *b*-values (0, 100, 600, 800)	50.3 ± 48.8	35.7 ± 43.7	0.60 [0.52, 0.68]	Reference
**Alternative 2-*b*-value combinations (Non-inferiority test)**				
ΔADC: *b* = 0, 600	47.1 ± 45.8	32.4 ± 40.9	0.60 [0.52, 0.68]	<0.001 ^a^
ΔADC: *b* = 0, 800	48.2 ± 47.5	34.1 ± 42.2	0.60 [0.52, 0.68]	<0.001 ^a^
ΔADC: *b* = 100, 600	55.9 ± 52.7	38.8 ± 45.8	0.61 [0.53, 0.69]	<0.001 ^a^
ΔADC: *b* = 100, 800	55.1 ± 53.2	39.3 ± 46.6	0.60 [0.52, 0.68]	0.006 ^a^
**Alternative diffusion metrics (Superiority test)**				
ΔADC_fast_ (0, 100)	19.2 ± 31.4	14.4 ± 30.3	0.54 [0.46, 0.62]	0.08 ^b^
ΔADC_slow_ (100, 600, 800)	55.3 ± 53.0	39.1 ± 46.4	0.60 [0.52, 0.68]	0.81 ^b^
Δ*f_p_*	–1.0 ± 44.1	4.7 ± 44.3 ^c^	0.56 [0.47, 0.64]	0.46 ^b^

^a^ *p* values for the non-inferiority test of AUC versus that of the reference ADC metric using all *b*-values, with a non-inferiority margin of 0.02 (*p* < 0.05/7 indicates non-inferiority after accounting for multiple comparisons). ^b^ *p* value for the superiority test of AUC versus that of the reference ADC metric using all *b*-values (*p* < 0.05/7 indicates superiority after accounting for multiple comparisons). ^c^ One non-pCR case was excluded from the Δ*f_p_* calculation due to a negative *f_p_* at the mid-treatment time point, attributed to noise or motion. Abbreviations: ADC, apparent diffusion coefficient; pCR, pathologic complete response; AUC, area under the receiver operating characteristic curve; CI, confidence interval; SD, standard deviation.

**Table 4:** Relative performance of two- versus four-*b*-value ADC calculations for predicting pCR stratified by cancer subtype.

Tumor ΔADC	pCR Mean ± SD (%)	Non-pCR Mean ± SD (%)	AUC (95% CI)	*p*-Value *
**HR+/HER2– (N = 88)**	**N = 15**	**N = 73**		
All *b*-values (0, 100, 600, 800)	75.1 ± 42.7	35.4 ± 39.6	0.76 [0.62, 0.89]	Reference
*b* = 0, 600	69.2 ± 41.2	32.2 ± 36.9	0.75 [0.62, 0.88]	0.003
*b* = 0, 800	72.2 ± 40.7	33.4 ± 37.6	0.77 [0.64, 0.89]	<0.001
*b* = 100, 600	84.7 ± 46.2	38.3 ± 40.6	0.78 [0.65, 0.91]	<0.001
*b* = 100, 800	84.9 ± 46.3	38.1 ± 40.7	0.77 [0.65, 0.90]	0.003
**HR−/HER2− (N = 65)**	**N = 24**	**N = 41**		
All *b*-values (0, 100, 600, 800)	32.7 ± 35.9	25.5 ± 39.6	0.57 [0.43, 0.72]	-
*b* = 0, 600	30.3 ± 33.9	24.0 ± 37.5	0.57 [0.42, 0.72]	-
*b* = 0, 800	31.3 ± 34.5	24.5 ± 38.5	0.58 [0.43, 0.72]	-
*b* = 100, 600	35.6 ± 39.9	28.6 ± 43.0	0.57 [0.43, 0.72]	-
*b* = 100, 800	35.6 ± 39.1	28.2 ± 43.2	0.58 [0.43, 0.72]	-
**HR−/HER2+ (N = 20)**	**N = 16**	**N = 4**		
All *b*-values (0, 100, 600, 800)	63.2 ± 64.7	35.0 ± 56.9	0.67 [0.27, 1.00]	-
*b* = 0, 600	60.2 ± 60.3	32.0 ± 52.8	0.67 [0.27, 1.00]	-
*b* = 0, 800	59.8 ± 63.5	34.1 ± 55.8	0.67 [0.27, 1.00]	-
*b* = 100, 600	69.3 ± 68.5	37.9 ± 60.2	0.70 [0.33, 1.00]	-
*b* = 100, 800	66.2 ± 69.1	38.9 ± 62.1	0.69 [0.31, 1.00]	-
**HR+/HER2+ (N = 37)**	**N = 15**	**N = 22**		
All *b*-values (0, 100, 600, 800)	39.8 ± 42.6	56.2 ± 56.3	0.56 [0.37, 0.75]	-
*b* = 0, 600	37.9 ± 39.4	48.9 ± 53.8	0.55 [0.36, 0.74]	-
*b* = 0, 800	38.8 ± 42.9	54.7 ± 55.2	0.57 [0.37, 0.76]	-
*b* = 100, 600	45.3 ± 44.5	59.3 ± 59.5	0.54 [0.35, 0.74]	-
*b* = 100, 800	44.8 ± 48.2	63.9 ± 60.9	0.57 [0.38, 0.76]	-

* *p* values for non-inferiority test of AUC versus that of the reference ADC metric using all *b*-values, with a non-inferiority margin of 0.02, calculated only for the HR+/HER2− subtype when the corresponding test for all cancers was rejected. Abbreviations: ADC, apparent diffusion coefficient; AUC, area under the ROC curve; CI, confidence interval; SD, standard deviation; pCR, pathologic complete response; HR, hormone receptor; HER2, human epidermal growth factor receptor 2.

**Table 5:** Test–retest repeatability of ADC, *f_p_* measures by different *b*-value combinations.

Metric	Mean ± SD ^a,b^	Limits of Agreement	wCV (%) (95% CI)
		Mean Difference ^b^ (95% CI)	
ADC: All *b*-values (0, 100, 600, 800)	1.17 ± 0.31	0.0097 [−0.1467, 0.1700]	5.36 ^c^ [4.60, 6.41]
**Alternative *b*-value Combinations, Metrics**			
ADC: *b* = 0, 600	1.22 ± 0.29	0.0085 [−0.1431, 0.1600]	4.94 [4.25, 5.91]
ADC: *b* = 0, 800	1.14 ± 0.28	0.0084 [−0.1446, 0.1600]	5.25 [4.51, 6.28]
ADC: *b* = 100, 600	1.13 ± 0.29	0.0085 [−0.1629, 0.1800]	6.01 [5.16, 7.19]
ADC: *b* = 100, 800	1.07 ± 0.28	0.0069 [−0.1597, 0.1700]	6.07 [5.21, 7.26]
ADC_fast_ (*b* = 0, 100)	1.76 ± 0.32	0.0094 [−0.3122, 0.3300]	6.71 [5.76, 8.02]
ADC_slow_ (*b* = 100, 600, 800)	1.08 ± 0.29	0.0072 [−0.1589, 0.1700]	6.01 [5.16, 7.19]
*f_p_*	0.09 ± 0.02	0.0009 [−0.0308, 0.0300]	12.37 [10.63, 14.80]

^a^ Averaged between test and retest measures. ^b^ ADC measures expressed as ×10^−3^ mm^2^/s; *f_p_* expressed as a fraction (scale 0 to 1). ^c^ wCV in this study was calculated following QIBA metrology guidelines [29] causing a difference from that reported in a prior paper (wCV = 4.8%; [12]).

## Data Availability

Data generated or analyzed during the study are available through ECOG-ACRIN, and all images and associated study metadata will be available on The Cancer Imaging Archive (TCIA) website (https://www.cancerimagingarchive.net, accessed on 10 January 2022), planned for public release in Spring 2022.

## Supplementary Materials

The following supporting information can be downloaded at: https://www.mdpi.com/article/10.3390/tomography8020058/s1, Table S1: Standardized ACRIN 6698 MRI Acquisition Parameters; Table S2. MR imaging systems utilized for the primary analysis and test-retest cohorts. File: Sample Size Estimates and Participating Institutions.

## Abbreviations

ADC, apparent diffusion coefficient; AUC, area under the receiver operating characteristic curve; CI, confidence interval; DCE, dynamic contrast-enhanced; DW, diffusion-weighted; IVIM, intravoxel incoherent motion; ROI, region-of-interest; ROC, receiver operating characteristic; NAC, neoadjuvant chemotherapy; wCV, within-subject coefficient of variation; pCR, pathologic complete response.
