# Supplementary material for: Impact of Alternate b-Value Combinations and Metrics on the Predictive Performance and Repeatability of Diffusion-Weighted MRI in Breast Cancer Treatment: Results from the ECOG-ACRIN A6698 Trial
Source: Tomography. 2022 Mar 4;8(2):701–17. doi: 10.3390/tomography8020058 (PMC8938828; doi:10.3390/tomography8020058)
Supplement: Supplementary file 1 [file tomography-08-00058-s001.zip › tomography-1571947-supplementary.pdf]

Supplemental Material

Table S1. Standardized ACRIN 6698 MRI Acquisition Parameters

Parameter	T2-weighted	DW-MRI	T1-weighted
**Sequence type**	FSE or STIR	DW SE-EPI	GE
**2D or 3D sequence**	2D	2D	3D
**Slice orientation**	Axial or sagittal	Axial	Axial
**Laterality**	Bilateral	Bilateral	Bilateral
**Frequency direction**	A/P	A/P	A/P
**Phase direction**	R/L (axial)<br>S/I (sagittal)	R/L	R/L
***FOV – frequency**	260-360 mm (axial)<br>180-220 mm (sagittal)	260-360 mm	260-360 mm
***FOV - phase**	260-360 mm (axial)<br>180-220 mm (sagittal)	300-360mm	260-360 mm
**Matrix – frequency (acquired)**	256-512	128-192	384-512
**Matrix – phase (acquired)**	≥ 256	128-192	≥ 256
**Reconstruction Matrix**	512 x 512	256 x 256	512 x 512
**In-plane resolution**	≤ 1.4 mm	1.7 – 2.8 mm	≤ 1.4 mm
**Fat-suppression**	Active fat-sat recommended	Active fat-sat	Active fat-sat recommended
**Repetition Time (TR)**	2000-10000 ms	≥ 4,000 ms	4-10 ms
**Echo Time (TE)**	70-140 ms<br>(STIR 70 ms)	Minimum	Minimum
**Echo Train Length**	≤ 16	N/A	N/A
**Inversion Time (TI; STIR sequence)**	170 ms (1.5T)<br>230 ms (3.0T)	N/A	N/A
**Flip Angle**	90 degrees	90 degrees	10-20 degrees
**Readout Bandwidth (per pixel)**	N/A	N/A	Maximum
***b*-values**	N/A	0, 100, 600, 800 s/mm^2	
**Slice thickness (acquired)**	≤ 4 mm	4-5 mm	≤ 2.5 mm
**Number of slices**	Variable; complete bilateral coverage	Variable; complete bilateral coverage	≥ 60; complete bilateral coverage
**Slice Gap**	≤ 1.0 mm	No gap	No gap
**Parallel imaging factor**	≤ 2	≥ 2	≤ 2
**No. of excitations or averages**	≤ 2	≥ 2	≤ 2

**k-space ordering**	N/A	N/A	-k to +k standard, non-centric
**Sequence acquisition time**	≤ 7 minutes	≤ 5 minutes	80 sec ≤ scan time ≤ 100 sec
**Total post-contrast imaging duration**	N/A	N/A	≥ 8 minutes following injection

Abbreviations: FSE = fast spin echo, STIR = short tau inversion recovery, DW = diffusion-weighted, SE = spin echo, EPI = echo planar imaging, GE = gradient echo, 2D = two dimensional, 3D = three dimensional, A/P = anterior-posterior, R/L = right-left, S/I = superior-inferior, FOV = field-of-view, N/A = not applicable

Table S2. MR imaging systems utilized for the primary analysis and test-retest cohorts.

	**Analysis Set N=210**	**Test-Retest Set N=71**
**Vendor**	**n (%)**	**n (%)**
**GE**	155 (73.8)	28 (39.4)
**Siemens**	30 (14.3)	12 (16.9)
**Philips**	25 (11.9)	31 (43.7)
**Field Strength**		
**1.5 tesla**	148 (70.5)	44 (62.0)
**3.0 tesla**	62 (29.5)	27 (38.0)

Sample Size Estimates

The sample size projection for the ACRIN 6698 trial was designed to achieve 90% power for the primary analysis to test whether the change in tumor ADC from baseline to the early treatment time point is predictive of pathologic complete response. Specifically, the sample size was calculated to detect a difference of 0.15 between the AUC under the null hypothesis of 0.5 and an AUC under the alternative hypothesis of 0.65 using a one-sided Z-test at a significance level of 0.05. It was

assumed that the number of pCR non-responders is approximately 2.7 times greater than the number of complete responders based on the ACRIN 6657 study, requiring a total of 160 analyzable patients (43 responders and 117 non-responders).

We assumed 20% of the cases accrued would not provide usable data for the analysis, for example, having missing MRI scans out of four MRI scans per participant, and assumed a 45% loss rate due to patients who are screened but do not proceed to the treatment phase of I-SPY 2. Additionally, 10% drop out rate is expected. Therefore, target enrollment was set at approximately 404 participants after accounting for the above three conditions.

Participating Institutions

University of California, San Francisco, CA

University of Washington, Seattle, WA

University of California, San Diego, CA

H. Lee Moffitt Cancer Center and Research Institute, Tampa, FL

University of Alabama, Birmingham, AL

University of Texas MD Anderson Cancer Center, Houston, TX

University of Chicago, Chicago, IL

Oregon Health and Science University, Portland, OR

University of Minnesota, Minneapolis, MN

University of Pennsylvania, Philadelphia, PA
